# Utility of Digital Technology for Ambulatory Assessment of Exposures and Health in Older Adults

**DOI:** 10.1093/geroni/igaf122.1886

**Published:** 2025-12-31

**Authors:** Carol Derby, Mindy Katz, Lesley Ross

## Abstract

Digital technologies including wearable censors and smartphones facilitate the real-time assessment of psychological and physiologic states, behavioral patterns, and cognitive functioning, as well as person-level environmental exposures in the course of daily life. Further, these methods allow for continuing follow-up of older adults when in-person assessments are not possible, such as during infectious disease outbreaks, or when older adults are not able to travel to a study clinic. This session will illustrate the application of digital technology to the study of cognitive aging within the Einstein Aging Study (EAS) cohort of community residing adults. The EAS utilizes an integrative approach that combines ecologically valid ambulatory measures of both exposures and cognition with traditional clinic-based assessments in a diverse community-based sample of adults aged 60 and above. The session will illustrate the feasibility and utility of this approach for: 1) maintaining ongoing follow-up in older adults and obtaining real-time longitudinal data during environmental events (Dr. Roque), 2) using GPS data to derive person-specific indices of neighborhood environment exposures as individuals navigate everyday life (Dr. Hyun), 3) understanding how glucose regulation impacts cognition in older adults with diabetes (Dr. Hoogendoorn), 4) examining real time associations between subjective cognitive concerns and cognitive performance (Dr. Garcia De La Garza), and 5) prospectively tracking psychological health during the COVID-19 pandemic (Dr. Wang).

